# Examining public private partnership investment in energy towards achieving sustainable development goal 7 for ASEAN region

**DOI:** 10.1038/s41598-024-66800-9

**Published:** 2024-07-16

**Authors:** Md Altab Hossin, David Alemzero, Hermas Abudu, Songtao Yin, Lei Mu, Boonsub Panichakarn

**Affiliations:** 1 School of Innovation and Entrepreneurship, Chengdu University, No. 2025 Chengluo Avenue, Chengdu, 610106 Sichuan People’s Republic of China; 2 School of Management and Economics, Southwest University of Science and Technology, 59 Qinglong Road, Mianyang, 621010 Sichuan People’s Republic of China; 3 College of Overseas Education, Chengdu University, No. 2025 Chengluo Avenue, Chengdu, 610106 Sichuan People’s Republic of China; 4 Department of International Cooperation and Exchange, Southwest University of Science and Technology, 59 Qinglong Road, Mianyang, 621010 Sichuan People’s Republic of China; 5 International e-Tourism Research Center, Chengdu University, No. 2025 Chengluo Avenue, Chengdu, 610106 Sichuan People’s Republic of China; 6 Faculty of Logistics and Digital Supply Chain, Naresuan University, 99 Moo. 9 Muang, Phitsanulok, 65000 Thailand

**Keywords:** SDG7, ARDL, Public–private partnerships investments, Energy, Southeast Asia, Energy access, Energy economics, Energy efficiency, Energy management, Political economy of energy

## Abstract

The gradual progress in aligning financial flows with the adoption of clean technologies reveals a persistent funding gap, signaling a global misallocation of capital. Addressing this challenge necessitates political leadership and robust policies to counteract the insecurities impeding the redirection of financial flows. This study investigates into the impact of energy-related public–private partnership investments (PPPIE) and macro-environmental variables on the attainment of Sustainable Development Goal 7 (SDG7) across Association of Southeast Asian Nations (ASEAN) member countries from 1999 to 2021. Employing the Dynamac command technique, we conduct autoregressive distribution lag analysis and the Bounds Cointegration Test to evaluate ASEAN’s efforts in achieving SDG7. Results indicate that a ten-year exogenous shock to the GDP growth rate initially causes a temporary decline in both GDP and PPPIE, albeit not statistically significant. However, in the long run, the shock becomes statistically significant, correlating with a negative decline in the GDP growth rate. This underscores the negative impact of external factors like the COVID-19 pandemic on the economic growth of ASEAN member countries. Specifically, a percentage increase in PPPIE leads to an 8.3% reduction in the GDP growth rate, revealing a detrimental and unsustainable impact on the economy. This signifies that energy investments in the ASEAN region, are predominantly unsustainable and adversely impact economic growth. Moreover, these energy investments contribute to a significant 52.6% increase in greenhouse gas emissions, indicating a substantial setback in the region’s progress towards meeting SDG7’s clean energy objectives by 2030. This suggests the present state of PPPIE does not align with sustainable clean energy goals of the region. Therefore, recommendations should include diversifying energy sources and investment strategies to enhance sustainable clean energy. Also, policymakers and researchers should reassess the terms and conditions of PPPIE, refining frameworks for private sector involvement to align with long-term economic sustainability goals.

## Introduction

Sustainable development goals seven requires a huge and combined financial obligation. If we want to achieve SDG7 by 2030, we must scale up investment yearly into electrification and clean cooking solutions. The amount required is not unrealistic given the amount of capital circulating worldwide. Yet energy investments are decreasing every year to achieve full electrification and unrestricted access to cooking solutions. Access to energy for industries and businesses is important to stimulate economic development. Likewise, household access to electricity is crucial to ensuring a fairer and healthier society. Without access to adequate finance, we essentially cannot achieve Sustainable Development Goal 7 (SDG7) by 2030. Achieving full global electrification by 2030 will require an annual investment of approximately $45 billion, with critical reliance on both public and private financing to close this financial gap^1^. In addressing the financial deficit, existing literature highlights the importance of private public partnership investments in various projects and solutions^2,3^. There is a resounding call for the private sector to take a leading role in mobilizing financing for the energy transition^4^. Public–private partnerships is characterized by collaboration between public and private entities to deliver public services or develop public assets with private investment^3,5^ and offer a strategic avenue for achieving this goal. Clean energy megaprojects often face challenges in securing sufficient funding from the public sector, given their substantial financial requirements. Introducing private sector participation becomes instrumental in unlocking additional financial resources necessary to actualize these large-scale initiatives and, in turn, accomplish SDG7. Furthermore, the advancement of financial mechanisms is indispensable for the effective deployment of negative emissions technologies^4,6^. Hence, it underscores the vital role of public investment in mitigating the gap caused by market dynamics. Public funding plays a crucial role in ensuring access to electricity for vulnerable communities and undeserved markets where the cost of electricity remains prohibitive.

According to literature, the public investment strategies and the right instruments need to be accelerated and implemented to achieve SDG7^7,8^.The need for public funding varies based on the type of technology, whether it is standalone, mini, or clean cooking solutions, as well as the stakeholders in the ecosystem, including end users and businesses. Access to tailored financing instruments is very important to achieve sustainable development and create an energy economy. Despite this, only 25% of the investment required for full electrification is Ref.^1^.Therefore, it is important to look at the region’s financial performance from the perspective of public–private partnership investments in energy (PPPIE) and green financing to achieve SDG7^9^. As a result, access to clean cooking solutions (ACKG) continues to lag as underinvestment and policy uncertainty make it difficult to achieve SDG7. To effectively determine policy options in this direction, this study aims to assess the performance of ten countries within the Association of Southeast Asian Nations (ASEAN) in achieving SDG7 via public private partnerships investment in energy. It is worth noting that energy demand in Southeast Asia is projected to grow by 60% annually by 2040, aligning with the region’s rapid economic expansion, population growth, and rising income levels^9^. It is undeniable that green finance and investments are crucial for realizing SDG7 in this context^10^. On the other hand, Baiashvili and Gattini^11^ believe that investments in the form of foreign direct investment are harmful to the environment. Importantly, the ASEAN region heavily relies on energy imports, accounting for approximately 40% of its energy requirements^12^. As the region experiences a surge in energy demand driven by population growth and economic development, the necessity of energy imports becomes increasingly evident^13^.

Furthermore, energy use is not sustainable in line with the United Nations SDG7, and therefore, it is imperative for the region to diversify its energy mix by including cleaner sources to meet the demand sustainably. More than 650 million people in the ASEAN region consume about 85% of fossil fuels^9,12^, resulting in an increase in emission levels of over 75%. In 2018, the installed power generation capacity was about 252 gigawatts (GW), and almost 30% came from renewable sources, mostly hydroelectric sources; in 2020, the share increased to 33.5% due to the expansion of photovoltaics. The ASEAN region also aims to achieve SDG7 and the Paris Agreement by 2030 by generating 23% of the primary energy supply and 35% of installed capacity from renewable sources^9^. Also, the region had only 14.3% of renewables in final primary energy consumption by 2021, which is below half in about a decade^12^. Meanwhile, the region has installed about 33.5 percent in a decade, a major increase in the last years^4,9^. According to the IRENA^9^, energy demand from the region increased by the mean in about a year and will continue until 2030 under their sustainable development scenario (SDS). Likewise, the ASEAN region nearly achieves universal access at almost 95% and ACKG solutions at 70%. The investment amount needed to achieve ACKG, and full electrification by 2030 is about $2.8 billion approximately, in a year^1,9^. Consequently, the achievement of SDG7 rests on the availability of critical minerals that may be successfully developed and improved capacity building in the region and investments in a wide range of areas. This even applies to the development of clean technologies and supply chains. For example, Indonesia, Philippines, and Myanmar are the first, second, and third largest nickel producers, accounting for 13% of the world’s rare earth minerals, and the ASEAN region supplies 6% of the world’s bauxite supply^14^.

Given the importance of the region to achieving SDG7, it is crucial to empirically determine this approach statistically to know the progress of the Southeast Asian nations. Here, with reference to Ref.^13^, the achievement of SDG7 was analyzed under the conditions of advanced economies and it was found that developing countries also depend on the current status of their policies and programs in order to achieve their goal^10^. Rightly so, Sakti et al.^15^ developed the novel spatial priority model for the development of renewables and discovered territories close to the equator (such as Indonesia, Malaysia, Brunei, and Singapore) have a lower potential for wind speed, which leads credence to the fact that wind speed potential is very high in the northern part of the region. As financing is important for the attainment of SDG7, Ahmed et al.^4^, Banga^10^ discovered the issuance of green bonds as an important policy tool for the deployment and development of renewables^16^. Green bonds will raise the needed finances for the development of- renewable energy projects. Between 2006 and 2016, Indonesia, Malaysia, Vietnam, Singapore, and the Philippines invested about twenty-seven billion dollars in clean energy development^4^. According to the RISE^17^, the Philippines grid investment is about $2 million a year between 2013 and 2017 due to overall investment fell sharply from $4.1 billion in 2015–2016 to $1.4 billion in 2017^9,18^. This makes affordability a challenge since 21.6% of the total population are living below the poverty line, and electricity prices in the Philippines are among the highest in Asia. After the formation of the Paris Agreement, financing for fossil fuel has increased from $612 billion in 2016 to $654 billion in 2018, making 33 global banks invest in fossil fuel to the tune of $1.9 trillion post the formation of the Paris Agreement^7,19^. Will this course lead to a reduction in fossil fuel financing? It is assumed that if fossil fuel financing decreases, investments in renewable energy will increase, other things being equal, and thus SDG7 will be achieved^13^. Therefore, the research question revolves around the Southeast region’s advancement in achieving SDG7 leveraging private–public investments. Specifically, have private–public partnership investments in energy augments achieving SDG7 in ASEAN Region? Thus, the study seeks to determine whether PPPIE contributes to the fulfillment of sustainable energy for the ASEAN Region.

This study presents a novel perspective by employing a distinctive methodology, utilizing a Dynamic Auto Regression Distributive Lag (Dynardl) model in conjunction with Auto Regression Distributive Lag (ARDL) bound cointegration to analyze investments in public–private partnerships within the energy sector. To assess cointegration, the study employs the pssbound as the post-estimation command, and the detailed results are provided in Table 1. Furthermore, the Dynardl model is utilized to simulate the impact of shocks on PPPIE and Gross Domestic Product (GDP), with the findings illustrated through graphs. This approach offers advantages, eliminating the need to make I (0)/I (1) differences for the regressors and ensuring the stationary nature of dependent variables and exploratory variables at the first difference. While various unit roots are available for cointegration testing, the Philip-Perron approach is chosen in this study. This method ensures that the variables are not of higher integration I (1). Although the ARDL Bounds test is relatively straightforward to implement, it necessitates specific critical values. In this study, we address this challenge by utilizing critical values provided by Jordan and Philips^20^, Silva et al.^21^ for asymptotic and finite sample analyses. This study is the pioneering effort in employing a Dynardl model in conjunction with ARDL bound cointegration to analyze investments in public–private partnerships in the energy sector within the Southeast Asian region. Additionally, a novel Dynardl model is introduced to simulate the impact of shocks on the variables. The novel results reveal that exogenous shocks exert long-term negative effects on both GDP and PPPIE, emphasizing the interconnectedness of economic and energy variables. Furthermore, the study finds that the reliance of countries in the region has led to a substantial increase in greenhouse gas emissions by over 52.6%. These novel findings underscore the urgent need for the ASEAN region to expedite its transition toward cleaner and sustainable energy sources by securing private sector financing. This approach is crucial to mitigating the environmental consequences associated with continued reliance on fossil fuels. Additionally, the research underscores the essential requirement for innovative solutions to achieve sustainable energy management in the region. The novel contributions of this study add to academic literature in its approach and findings towards enhancing sustainable energy in ASEAN region.

**Table 1:** Pesaran, Shin and Smith^67^ cointegration test.

Test name	Values					Stat
F-test	< ----- I (0) ---------- I (1)----- >					1.844
	10% critical value		2.578		3.858	
	5% critical value		3.125		4.608	
	1% critical value		4.537		6.370	
t-test	< ----- I (0) ---------- I (1) ----- >					0.239
	10% critical value	− 2.570		− 3.860		
	5% critical value	− 2.860		− 4.190		
	1% critical value	− 3.430		− 4.790		

Small-sample critical values not provided for t-statistic; asymptotic critical values used for case III.

The rest of the research is organized and structured as following sections. Section “Literature review” represents an overview of the literature on related areas. Section “Methodology” deals with methodology. Section “Results and discussion” discusses the results. Finally, Sect. “Conclusion, policy recommendation, and limitation” summarizes the study with the conclusion.

## Literature review

The ASEAN region’s achievement of SDG7 hinges on the availability of financial resources to implement programs and policies geared toward the realization of SDG7 within the midterm to the long term. This aspect reviews the literature in relation to the financial availability and the realization of SDG7 alongside other indicators. According to IRENA^9^, Vakulchuk, et al.^13^, the attainment of the 1.5-degree scenario is feasible and can cut costs aggregately connected to energy supply at about $160 billion by 2060^1,8^. Furthermore, avoided externalities emanating from the 1.5-degree scenario range from $508 billion to $1580 billion aggregately to the midterm century^9,22^. The achievement of SDG7 depends on finances as well as the critical minerals (CRMs) that will be raw materials for the manufacturing of solar parts plus wind energy turbines. As a result, environmental, social, and governance (ESG) issues are of prime concern for the region^23^. The Southeast Asian region, if it could develop its internal value chains for different industries, earnings from tin, rare earth minerals, and nickel could increase by more than double to $60 billion by the midcentury in the SDS^22^. Therefore, improving the capacity development of this region is crucial to developing these minerals sustainably and attracting private investments^24^. This capacity development could be from technical and non-technical assistance^25^. A study by Sarpong et al.^26^ discovered that a one percent increase in renewable energy consumption reduced carbon emissions by 0.144%, while an increase in economic growth increased emissions by 0.961%. This was done for the seven emerging markets (E7) using feasible generalized least squares (FGLS). A recent study by Durani et al.^27^ using second-generation unit root tests and panel quantile regression for the period 1990–2020 in BRICS economies to assess whether economic policy uncertainty moderates the association between renewable energy and carbon emissions. They found that renewable energy and economic policy uncertainty reduce carbon emissions at all quantiles, while financial development and employment levels increase them. Likewise, Jahanger et al.^28^ applied the moment method of quantile regression (MMQR) for BRICS between 1990 and 2018 using the Kuznets curve theory of the environment. Their results showed that renewable energy and natural resources can effectively curb environmental degradation. Additionally, energy use and GDP per capita in the region have increased significantly, with Singapore having a GDP per capita of $100 Thousand USD, followed by Brunei Darussalam, Malaysia, Thailand, Indonesia, Vietnam, Philippines, and that order. Cambodia is the least in GDP per capita of under $20 Thousand USD (2019, PPP)^9,12,18^. On the other hand, energy demand per capita is growing substantially in the region due to its increasing population^12^. Brunei Darussalam is leading the region with 2000 per joule (PJ) of energy demand per capita, next to Singapore, and Myanmar is the least^9,12^. The Southeast Asian region’s population grew about 10% in a decade, bringing the population to over 660 million people, and the economy expanded around 4.2% in the mean level between the periods 2010–2019^29^. Correspondingly, the Southeast Asian region has coal, together with fossil fuels, as the dominant fuel, with renewable use increasing twice since the 2000s^29^. Also, the use of biomasses in households for cooking and heating was reduced by half^30^. For instance, solar photovoltaic (PV) is gaining ground due to strong policy support and low-cost auctioning^9^. More so, minimizing local content and ownership rules and making standard public–private agreements (PPAs) viable will attract international investments^13^. Renewable capacity in the Southeast Asian region is anticipated to increase to 51 GW during 2022–2027, over a 56% growth rate. Solar PV will make up more than half, next to onshore wind and hydropower^7,15^. As a result, solar PV is expected to lead the increase and accelerated deployment due to strong policy advancements and robust procurement processes as well as auction schemes in Indonesia and the Philippines^15^.

**Figure 1:**
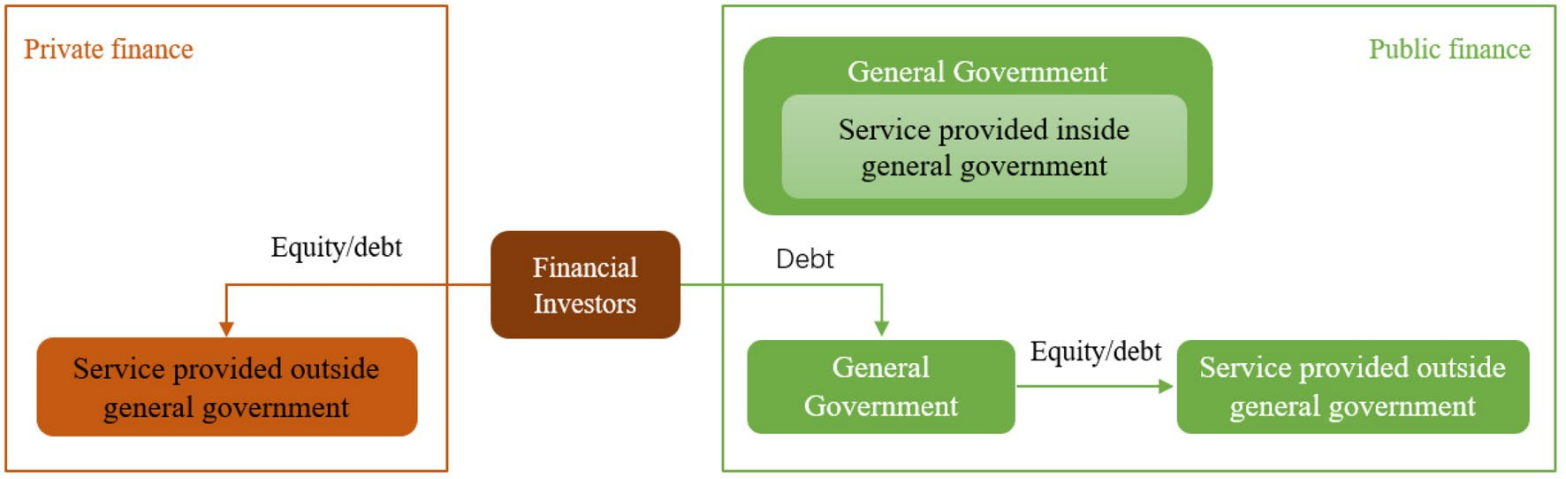
Conceptual framework. *Source*: Eyraud et al. ^51^.

Figure 1 illustrates that the realization of the sustainable energy development that hinges on SDG7 depends on the availability of the necessary finance in both the private and the public sectors to scale the development and deployment of renewables^31^. Emmerling, et al.^32^ agreed that joint commitments are needed to increase scaling up financing for the SDGs by putting sustainability practices in financial decision-making and implementation and that the SDGs^33^, especially SDG7, need to be at the Centre-of-the-road in public financial decision-making^1,34,35^. Additionally, environmental innovation is very key in unleashing novel ideas for new technologies, especially in clean energy deployment that curtails environmental degradation^36^. This will bring diffusion of knowledge and skills across the region. Indeed^37,38^, using an advanced econometric model to analyze the energy transition and environmental innovation of the top ten manufacturing countries concurred with the notion that financial development is central to industrialization, its downside risks of ecological and environmental consequences cannot be overlooked as it leads to economic growth and over energy consumption^38^. Generation of energy from clean energy sources can reduce carbon footprint since the electricity sector is the greatest emitter of carbon dioxide that causes temperature increase^39^. Consequently, Ahmed, et al.^4^ found that innovations in the development of green technologies and the consumption of renewable energy reduce the carbon emissions of the twelve emitters of carbon emissions using advanced econometric models such as the second-generation unit root test and the Demetricus and Hurlin (D-h) causality test between 1991 and 2018. Green innovation is very central to abating carbon emissions from medium to higher quantiles. This was discovered from Ref.^4,40,41^, where they theoretically used the Stochastic Impacts by Regression on Population, Affluence, and Technology (STRIPAT) model deploying the method of moment quantile regression (MMQR) between 1991 and 2018. Likewise, Durani et al.^27^ applied quantile regression heterogeneously to assess the E7 economies in terms of technological innovation, institutional quality, per capita income and carbon emissions, and documented that technological innovation played an important role in reducing carbon emissions in the E7 between 1996 and 2018.This calls for the modernization of the grid to take on renewable sources by upgrading the infrastructure. Infrastructure development in the form of transport from the 60th to the 90th quantile contributes to sustainable development through environmental innovations that reduce emissions from the transport sector. This was achieved through public–private partnerships (PPP), as found by Ref.^27,42^ using a quantile ARDL method. Regarding generation, it is anticipated that the region has to spend a percentage of its GDP in order to attain if they are to achieve the 23% of renewable installations, which translates to $27 billion yearly and $290 billion by 2025^9,13^. According to Siddik et al.^43^, higher GDP per capita can encourage renewable energy (RE) consumption and production, which indicates that developed country countries have mature energy markets^44^. Thus, richer countries can develop their energy systems much faster than poorer countries^45^. According to White et al.^46^, the Asia Pacific, apart from China, attracted $213 billion in energy supply investment and facilitation in 2021; $98 billion was invested in clean energy sources, and $115 billion was invested in fossil fuel sources^46^. This narrative denotes the ASEAN region as heavily dependent on fossil fuel sources. Similar work has been carried out by Ref.^10,47^, where they decried the slow pace of green bonds in the emerging markets unlike the advanced world, and that the development of green bonds remains nascent and underexplored and bemoaned the lack of suitable institutions for the management of green bonds and high transactional costs as the limitations to the growth of green bond in emerging countries. Standalone and renewable investment in Southeast Asia was reduced by 98%, making more off-grid companies fragile. Despite the fact is that the region nearly attains universal access, Myanmar and Cambodia are yet to attain full electrification^9,13,48^. This fall in investment further dampens their ability to achieve SDG7. In addition, the region attracted US$137 million in investments in off-grid and renewable energy in 2018–2019, of which Myanmar received a larger percentage^29,48^. Comparably, Haas and Popov (1990), in studying the Organization for economic cooperation and development countries (OECD), revealed that financial development promotes economic growth and urges carbon-intensive sectors to develop and implement cleaner generation sources^49^.

Likewise, Ahmed, et al.^4^, Samour et al.^50^ discovered in their study of six Southeast Asian countries using the STIRPAT (Stochastic Impacts by Regression on Population, Affluence, and Technology) model approach and sighted green bonds are effective in promoting green energy projects and in reducing carbon dioxide emissions. From the conceptual framework in Fig. 1, private finance refers to stake or equity emanating from institutions other than the state for the development of projects. A private entity’s liabilities are not covered by the state. Thus, private entities don’t grow government debts. A case in point is that a person buying a government bond can be considered public financing while financing from multilateral development finance institutions is considered private financing as it does not increase government debt^51^. On the other hand, public financing is the situation in that development projects are explicitly or implicitly funded by the government, thereby affecting the liability of the state^51^. From 2010 and 2021, debt and equity investments were made up of 47% and 48% of cumulative financing, with an extra 5% contribution from grants^52^. The study further noted that equity finance was mainly used to finance company expansion and growth. Equity was used to fund the mini-grid and off-grid, accounting for over 74% of the total funding. While most of the technology investment in solar lights in 2010–2021 was financed with debt at around 54%^9,13^.

In summary, previous research in this area concluded that the achievement of SDG7 rests on the availability of private and public sector investments to accelerate the attainment of SDG7 and that green investments have environmental benefits in this regard for the Southeast Asian region. However, the gap is that the Southeast Asian region has not developed the necessary framework to attract private investment and the public sector still invests heavily in fossil fuels, more than in renewable energy. As a result, green energy projects are underfunded, jeopardizing the achievement of SDG 7. Therefore, it is necessary to study this aspect to offer solutions for scaling strategies to achieve SDG7 for this region using public–private partnership investments.

## Methodology

The study will utilize the simulation of auto regressive distributive lag (ARDL) models in carrying out this research that will evaluate the investment performance of the ASEAN economies in achieving SDG7. In doing this, we seek to measure the indicators and other macroeconomic variables, such as the GDPRATE, that are characterized by a sizable number of complexes of lag structures, contemporaneous values, first differences, lagged first difference of independent variables in the models. The data for the indicators were derived from the World Bank Development Indicators (WDI). Because it is sometimes difficult to explain one lag in the dependent variable and the other lag in the independent variable and their long-term and short-term effects, it can be difficult to average those effects. In view of this, we will make use of the dynardl estimate that is able to approximate in Stata and determine dynamically the ARDL-bounds cointegration error correction model in conducting how investment in energy by the private and public (namely, PPPIE) sector can contribute to achieving the SDG7. The ARDL model can derive estimates from the data, store results, and graph key predictions. The analysis can provide a visual impression of the counterfactual variations in a regressor at a given point in time without varying the other variables using stochastic variation approaches. Before conducting the analysis using this approach, we know that the dynamic simulation method is gaining traction among researchers. This is due to the ability to represent important key results of time series models whose coefficients have no hidden meaning^53,54^. As a necessary condition for the determination using this approach, we determined the order of integration of the variables via the Fisher-type unit-root test based on Phillips–Perron tests at a level. As a result of an error correction model in the analysis, we use the Pesaran, Shin, and Smith Cointegration (pssbound) as a post-estimation to evaluate for cointegration in the model instead of specifying an additional model.

### Theoretical model

We theorize the SDG7 indicators in relation to the ASEAN region and anchor the analysis as one of the key indicators as an explained parameter, namely finance, which is a proxy for private public partnership investments in energy. Since this is a macro of a structural study, we use the ARDL model. Therefore, we specified the general ARDL model $\left( {\rho ,q} \right)$ in which a variable $y_{it}$ is a function of a constant term $\left( {a^{0} } \right)$ previous values of itself reaching back to a phase $q$ years, simultaneous and past values of the explained parameter $x_{it}$ of lag order $q$ with an independent, identically distributed stochastic term, as done in the study of Philips^53^ The variables to be analyzed are presented in Eq. (1) before the ARDL model. Equation (3) is the fixed effect form of the variables. From the model and Eq. (2), PPPIE is the dependent variable that explains public–private partnerships’ investment in energy in current United States dollars. This is an important variable regarding how the Southeast Asian region can achieve its SDG7. Also, ACKG denotes excess to clean cooking solutions and is one of the pillar indicators of SDG7. Globally, a lot of people die from indoor and outdoor pollution because of using polluting sources^55–57^. EI explains the amount of energy used to produce one unit of GDP. It is a measure of each country’s level of efficiency^58^. In addition, the renewable energy output shows the amount of renewable energy produced from the final electricity generation. The region aims to generate 23% of renewable energy in final energy consumption by 2025^59^. We will analyze this variable to see if this is achievable. The GDP rate reflects the pace of development in all areas of the economy. It is assumed that the higher the GDP, the more countries can invest in achieving SDG7^60–62^. Human activities cause the carbon emissions that bring about global warming and its associated consequences. The study will assess how the Southeast Asian region is progressing towards meeting this goal of reducing global warming GHGs, with electric vehicles able to reduce GHGs by up to 20% when powered by renewable energy sources^63–65^.

1 $$F\left( {PPPIE, ACKG,EI,REO,GDPRATE,GHG} \right)$$

2 $$lnPPIE _{it} = a^{0} + \beta_{0} lnACKG _{it} + \beta_{1} lnEI_{it}^{ } + \beta_{2} lnREO_{it} + \beta_{3} lnGDPRATE _{it} + \beta_{4} lnGHG _{it} + \varepsilon_{it}$$

3 $$y_{it = } a^{0} + \mathop \sum \limits_{i = 1}^{p} a_{i} y_{t - 1} + \mathop \sum \limits_{j = 0}^{q} \beta_{j} x_{t - 1} + \varepsilon_{it}$$

Here $\varepsilon_{it\sim } {\text{\rm N}}\left( {0,\delta^{2} } \right)$, the stochastic time. From Eq. (3), the data generation process as we determine it, is to make sure the explained and exploratory variables are stationary or without unit roots. If the variables are stationary on either side of the model, that means they exhibit equal mean, variance, and covariances, then the ARDL model in ARDL $\left( {q,\rho } \right)$ Eq. (3) is used. Due to the fact of the addition of many lags will cause multicollinearity, we set the restriction on ARDL $\left( {1,1} \right)$ model.

4 $$y_{it = } a^{0} + a_{i} y_{t - 1} + \beta_{o} x_{t} + \beta_{0} x_{t - 1} + \beta_{o} x_{2} + \beta_{0} x_{t - 2} + \beta_{o} x_{3} + \beta_{o} x_{t - 3} + \ldots + \varepsilon_{it}$$

### Empirical model

We then fit the model to ascertain the estimation of the explained variable in levels using Eq. (4). At the same time, Eq. (5) represents the variables based on Eq. (4).

5 $$\begin{aligned} lnPPPIE_{it} & = a^{o} + lnPPIE_{t - 1} + lnACKG + lnACKG_{t - 1} + lnEI + lnEI_{t - 1} \\ & \quad + lnREO + lnREO_{t - 1} + lnGDPRATE + lnGDPRATE_{t - 1} \\ & \quad + lnGHG + lnGHG_{t - 1} + \varepsilon_{it} \\ \end{aligned}$$

The contemporary impact of $x_{it}$ on $y_{t}$ is given by $\beta_{0}$. The scale of $a^{0}$ denotes the memory of $y_{it}$ ^20^. We assume that $0 < a^{0} < 1$, bigger values imply that the effect of movement on $y_{t}$ takes time to die off.

The generalized error correction model (GECM) is applicable since all the variables are stationary. The most widely applied GECM is the one-step GECM, which is statistically expressed to be equal to ARDL $\left( {1,1} \right)$ model^20,21,53,66^.

6 $$\begin{aligned} \Delta y_{it} & = a^{0*} + a_{i} y_{t - 1} + \beta_{o} \Delta x + \beta^{*}_{1} x_{t - 1} + \beta \Delta_{0} x_{2} + \beta_{2}^{*} x_{t - 2} + \beta_{0}^{*} \Delta x_{t} \\ & \quad + \beta_{t - 3}^{*} + \beta_{0} \Delta x_{t} + \beta_{4}^{*} x_{t - 4} + \beta_{0} \Delta x_{ } + \beta_{5}^{*} x_{t - 5} + \varepsilon_{it} \\ \end{aligned}$$

where the first difference of $y_{it}$ is a function of the constant term $a^{0}$, its own lag. $y_{t - 1}$ is the first difference of $x_{t}$ and its lag $x_{t - 1}$, plus identically independently distributed noise term $\varepsilon_{t}$. Even though it is statistically equal to ARDL $\left( {1,1} \right)$, the explanation changes using GECM models vary. From the model, the changes of $y_{t}$ on $x_{t}$ are given by $\beta_{0}$. The level of adjustment or pace of aggregate impact of variation of $y_{it}$ on $x_{t}$ is given by $a_{1}^{*}$ in Eq. (6).

### ARDL bounds test

The ARDL-bound model test by Pesaran and Shin are given below in Eq. (7).

7 $$\begin{aligned} y_{it} & = a^{0} + \emptyset_{yt - 1} + \emptyset_{1} x_{1t - 1} + \cdots + \emptyset_{k} x_{kt - 1} + \mathop \sum \limits_{i = 1}^{p} a_{i} \Delta y_{t - 1} \\ & \quad + \mathop \sum \limits_{j = 0}^{q1} \beta_{j} \Delta x_{1t - j} + \cdots + \mathop \sum \limits_{j = 0}^{qk} \beta_{j} \Delta x_{kt - j} + \varepsilon_{it} \\ \end{aligned}$$

The number of lagged first difference $y_{t}$ on $x_{t}$ are determined by the SIBC simulation. Post the estimation, the F-test of the null hypothesis is conducted that $\emptyset_{0} = \emptyset_{1} \ldots 0_{k} = 0$. The pssbound command was analyzed to test for cointegration according to Jordan and Philips^20^, Pesaran et al.^67^. Table 2 encapsulates the variables used in this study and their corresponding definition.

**Table 2:** Definition of variables. *Source*: https://databank.worldbank.org/source/world-development-indicators.

Variable	Definition
Public–private partnerships investment in energy (current US$)	Public–private Partnerships in energy (current US$) is the value of commitments to energy projects that have reached financial closure and directly or indirectly serve the public, including lease and management contracts, operation and management contracts with major capital expenditure, and greenfield projects (in which a private entity or public–private joint venture builds and operates a new facility)
Access to clean fuels and technologies for cooking (% of the population)	Access to clean fuels and technologies for cooking is the number of the aggregate population mainly using clean cooking fuels and technologies for cooking (kerosene is excluded as per the world health organization (WHO) guidelines)
Energy intensity level of primary energy (MJ/$2017 PPP GDP)	Energy intensity level of primary energy is the ratio between energy supply and gross domestic product measured at purchasing power parity. Energy intensity is an indication of how much energy is used to produce one unit of economic output
Renewable electricity output (% of total electricity output)	Renewable electricity is the share of electricity generated by renewable power plants in total electricity generated by all types of plants
GDP growth (annual %)	Annual percentage growth rate of GDP at market prices based on constant local currency
Total greenhouse gas emissions (KT of CO2 equivalent)	Total greenhouse gas emissions in KT of CO2 equivalent are composed of CO2 totals excluding short-cycle biomass burning (such as agricultural waste burning and savanna burning) but including other biomass burning (such as forest fires, post-burn decay, peat fires and decay of drained peatlands)

*PPP*: purchasing power parity.

## Results and discussion

### Descriptive and preliminary results

This section presents the discussion of the study. The descriptive statistics in Table 3 show that the PPPIE has the highest mean value. This indicates that, on average, the region performs well when it comes to public–private energy investments. Its maximum value is 22.496, and its minimum value is 15.226. On the other hand, total GHGs are the second-highest mean variable. It is no surprise that this variable reaches this level as the region has some of the largest emitters, and this is reflected in the region’s worsening hurricane climate conditions. This means that regions need to do more in terms of climate adaptation and mitigation^10,68^. Additionally, ACKG receives the second-largest mean, implying the region’s progress toward SDG7. Some of the ten countries have achieved this indicator from the SDG7 of universal ACKG, such as Singapore. Some countries are close to universal access. The fourth variable is renewable electricity output (REO). This denotes progress in the ASEAN region on this indicator but at a gradual pace. Sustainable energy can be achieved through renewable generation and consumption, as the region has the target to reach 23% of renewable energy production by Ref.^9,69^. It is important to mention that the gross domestic product growth rate (GDPRATE) gives the second smallest mean. This explains the sublime growth rate of national economies. The last variable with the smallest mean is energy intensity (EI). This indicator of SDG7 explains the efficiency of a country in its development efforts. The data shows that the least developed countries are those with worse EI^69^. Cambodia has the worst EI in double digits, supporting the claim that the least developed countries are financially unable to modernize their energy systems^12^.

**Table 3:** Descriptive statistics. *Source*: Authors’ estimation.

Variable	Obs	Mean	Std. Dev	Min	Max
lnPPPIE	98	19.742	1.802	15.226	22.496
lnACKG	189	3.495	1.239	0.262	4.605
lnEI	181	1.568	0.481	0.718	3.605
lnREO	140	2.524	1.8	− 3.115	4.605
lnGDPRATE	191	1.577	0.852	− 2.952	2.676
lnGHG	189	11.165	1.294	8.734	13.017

ln: natural logarithm function of the variables.

Tables 4 and 5 show the Fisher type unit root test. Based on Phillips–Perron single level and first difference testing. While’s Table 6. Dynamic estimates of fixed panel models show the main analysis of the study in which the fixed panel models are analyzed with different ARDL models. Table 1. The cointegration test is a post-command estimate for cointegration analysis^20^. Figure 4 is created to determine how well an estimation model fits the data and to identify the influencing relationship between variables. Table 7 presents the correlation matrix, illustrating the relationships among the variables. Notably, the most significant correlation is observed between total GHGs and ACKG. The correlations range from negative to positive, reflecting delicate associations. PPPIE exhibits a perfect correlation of one. REO demonstrates a positive and ascending correlation with PPPIE. On the other hand, EI exhibits a negative correlation with PPPIE and most variables, signaling a declining trend in EI and, to some extent, the region’s efficiency. This implies that nations investing in sustainable energy projects enhance their efficiency. The decreasing or negative relationship signifies a structural shift, highlighting investments in energy conservation, efficiency, and demand-side control. A noteworthy correlation exists between GHGs and REO, which is negative. This indicates a reduction in total GHGs as REO increases. This aligns with the fact that REO does not emit carbon dioxide and other GHGs contributing to climate change. Therefore, to curtail greenhouse gas emissions, the Southeast Asian region must amplify its utilization and development of renewable energy, as the relationship is inverse.

**Table 4:** Fisher-type unit-root test Based on Phillips–Perron tests at a level. *Source*: Authors’ estimations.

Variable	*P*-value	Lag(s)	Time trend
lnPPPIE	0.0005	1	Included
lnACKG	0.00	1	Included
lnEI	0.6285	1	Included
lnREO	0.393	1	Included
lnGDPRATE	0.00	1	Included
lnGHG	0.9847	1	Included

**Table 5:** Fisher-type unit-root test Based on Phillips–Perron tests first difference. *Source*: Authors’ estimations.

Variable	*P*-value	Lag(s)	Time trend
lnPPPIE	0.00	1.00	Not included
lnACKG	0.00	1.00	Not included
lnEI	0.00	1.00	Not included
lnREO	0.00	1.00	Not included
lnGDPRATE	0.00	1.00	Not included
lnGHG	0.00	1.00	Not included

**Table 6:** Dynamic fixed panel model estimations. *Source*: Authors’ estimations.

Variable DV: lnPPPIE	Model 1(dynardl)	Model 2 (dynardl)	Model 3 (DFE)	Model 4 (FE)
Main				
L1.lnPPPIE	− 0.804***	0.226		
	(− 4.05)	− 0.24		
D.lnGDPRATE	− 1.121	4.029		
	(− 1.39)	− 1.12		
L1.lnGDPRATE	− 0.759	11.86		
	(− 0.73)	− 1.39		
D.lnACKG	− 2.228			
	(− 0.15)			
D.lnEI	− 12.91	14.7		
	(− 1.24)	− 0.69		
D.lnREO	− 1.383	− 6.455		
	(− 1.04)	(− 0.67)		
D.lnGHG	24.86	18.31		
	− 1.91	− 0.69		
L1.lnACKG	− 1.782	− 13.77		
	(− 0.96)	(− 1.57)		
L1.lnEI	− 1.067	11.15		
	(− 0.50)	− 1.07		
L1.lnREO	− 0.418	− 5.835		
	(− 0.56)	(− 2.33)		
L1.lnGHG	1.504	9.931		
	− 1.08	− 1.75		
L1D.lnPPPIE		− 1.35		
		(− 1.82)		
L1D.lnACKG		− 42.92		
		(− 0.77)		
L1D.lnEI		− 18.44		
		(− 0.91)		
L1D.lnREO		− 14.31		
		(− 1.58)		
L1D.lnGDPRATE		− 3.353		
		(− 1.60)		
L1D.lnTGHG		− 64.09		
		(− 1.10)		
lnACKG			− 3.441	− 1.059
			(− 1.07)	(− 0.87)
lnEI			− 0.556	2.89
			(− 0.14)	− 1.92
lnREO			− 1.896	− 0.235
			(− 0.77)	(− 0.43)
lnGDPRATE			− 1.332	− 0.920*
			(− 1.18)	(− 2.22)
lnGHG			7.438*	4.607*
			− 2.37	− 2.56
_cons	7.788	− 85.98		− 32.63
	− 0.65	(− 1.26)		(− 1.77)
SR				
ec			− 1.043***	
			(− 4.86)	
D.lnACKG			9.676	
			− 0.58	
D.lnEI			− 7.438	
			(− 0.64)	
D.lnREO			− 1.111	
			(− 1.06)	
D.lnGDPRATE			0.211	
			− 0.35	
D.lnGHG			15.3	
			− 1.09	
_cons			− 50.61	
			(− 1.47)	
N	39	23	64	64

*DFE*: dynamic fixed effects; *FE*: fixed effects.

p*** > 0.001, p** > 0.01, p* > 0.05.

**Table 7:** Correlation matrix. *Source*: Authors’ estimations.

Variables	lnPPPIE	lnACKG	lnEI	lnREO	lnGDPRATE	lnGHG
lnPPPIE	1					
lnACKG	0.093	1				
lnEI	− 0.129	0.189	1			
lnREO	0.235	− 0.313	− 0.529	1		
lnGDPRATE	− 0.294	− 0.486	− 0.049	0.171	1	
lnGHG	0.109	0.946	0.159	− 0.223	− 0.43	1

As the necessary requirement to estimate an error correction ARDL approach, the variables must be stationary. So, testing for co-integration is vital, as well as the stationarity of the variables. The fisher-type-unit root test, based on Philips–Perron tests at level, was carried out with one lag and time trend included. As shown in Table 4, some of the variables are stationary, and others do have unit roots. The next table (Table 5) depicts the results at first difference.

In Table 5, the variables were differenced at the first level, demonstrating stationarity at the first difference I (1). This allows for the subsequent analysis of the ARDL model.

### Empirical model results

From Table 6, model 1 is the dynardl analysis. The dependent variable is depicted as a lagged variable but also as a lagged difference. We equally added a lag difference of one for all variables and lagged $$t - 1$$ for each of the variables. Whereas diff $$\left( {,1,1,1} \right)$$ for the remaining explanatory variable, enter the model as the first difference. From model 1, the first lag of PPPIE is significant demonstrating the relevance of investment in the ASEAN region to attain the SDG7. This result is confirmed by Ref.^4,52,69^ who revealed that the Global Climate Fund (GCF) approved an investment portfolio of $795 million in 2021 to support the region’s investment efforts. In addition, the GCF ASEAN Catalytic Green Finance Facility (ACGF) was recently implemented by the Asian Development Bank, which is a public–private co-investment initiative designed to simultaneously support a broad portfolio of green infrastructure to achieve sustainable development and to enable an inclusive economic recovery in the Southeast region.

**Figure 2:**
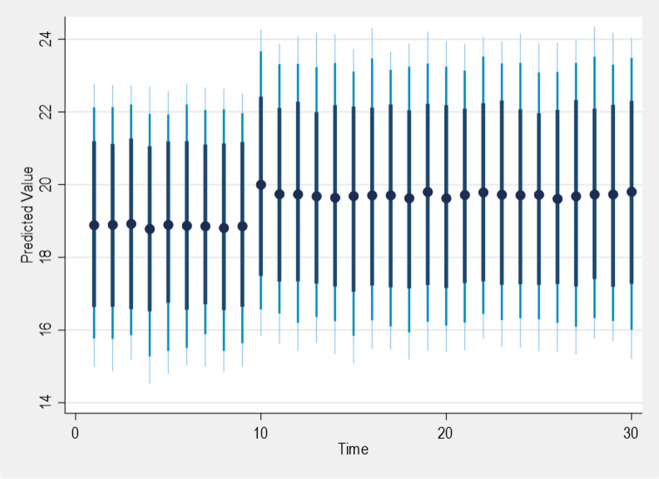
Plot generated by Dynardl.

Regarding a shock term in Fig. 2 below, we specify the variable to be shocked as GDPRATE and the amount of time to be shocked and added options such as the time range of ten for the shock to occur, thirty include the amount simulation, and “ec” to include the dependent variable. Finally, we indicate the graph option for it to occur. Dynardl will generate a graph, and below is the intuition behind the results. From graph one, a negative shock at $$t = 10$$ will cause a reduction in GDPRATE and PPPIE, but not statistically significant in the short run, as depicted in Fig. 2. In contrast, Fig. 2 represents, in the long term, the shock will cause a negative increase to almost eight, which is significant. Amidst the COVID-19 pandemic, we witnessed a substantial decrease in green financing, marked by a decline in sustainable funding from both public and private sectors. Despite these challenges, renewable energy displayed remarkable resilience, experiencing growth in electricity supply. Notably, countries like China and the US spearheaded this progress, anticipating a 4% global increase in net renewable energy installations, reaching approximately 200 GW in 2020. The aftermath of these shocks saw large-scale wind and hydropower contributing to almost 90% of the additions in 2020. In response to the economic downturn, governments worldwide initiated $460 billion in short-term energy packages to revitalize their economies, aligning with the “build back better” (BBB) initiative. Furthermore, the International Monetary Fund (IMF) played a crucial role by providing special drawing rights (SDRs) to aid emerging countries in sustainable recovery from the pandemic. Of the total, $109 billion is allocated to foster economic growth through sustainable energy development and utilization. The ASEAN economy, grappling with pandemic-related disruptions, contracted by 4% in 2020 but showed signs of recovery with a 3% growth in 2021. The ongoing pandemic significantly impacted energy demand, leading to a 3% decline in the entire energy supply chain. Notably, oil suffered the most, plummeting by 6.5%, given the region’s substantial oil consumption^70,71^.

In the dynamic fixed-effects model 3, only GHGs exhibit significance for PPPIE. The significance and the coefficient direction suggest that PPPIE contributes to increased GHG levels in the Southeast Asian region. This finding is corroborated in the study of Hoa et al.^12^ who documented that the Asia–Pacific region is a global player in terms of energy consumption and represents a major source of greenhouse gas emissions, accounting for 50% worldwide. Furthermore, about 75% of these greenhouse gas emissions come from energy consumption. This aligns with the region’s higher reliance on fossil fuels, particularly coal, as highlighted_ENREF_72^72^. In 2021, the Asia–Pacific region (excluding China) directed $213 billion toward energy financing and development, with $115 billion allocated to fossil fuels and $98 billion to clean energy^7,9,48,73^. Given the region’s over-reliance on coal, two out of four plausible options for ensuring a just transition have been proposed, based on reducing fossil fuel consumption and investing in renewable energy^72^. Consequently, Naumann et al.^74^ confirmed that coal-based energy production resulted in greater environmental degradation than gas-generated energy and that renewable energy reduces environmental degradation and improves environmental sustainability.

Lastly, Model 4 entails a fixed effects analysis, aiming to address the country-specific effects in factors influencing green financing in the ASEAN region. Model 4 has the log of Greenhouse gases (GHGs) and the log of GDP growth rate being significant. Particularly, the GDP growth rate exhibits significance but with a negative correlation. Given that the region heavily relies on fossil fuels for more than 80% of its energy consumption, this negatively impacts economic development, leading to a reduction in the GDP rate by up to 8.3%. Moreover, the ASEAN region experiences a noteworthy increase in greenhouse gas emissions, reaching up to 52.6% (ln 4526), due to the significance of the GHGs variable. Also, Jiang et al.^42^ showed that institutional quality promotes the use of renewable energy and gross domestic product (GDP) has a positive impact on the environment. In addition, Liu et al.^29^ supported this finding by noting that CO2 emissions in emerging markets depend on the GDP growth rate, energy consumption and other macro variables. Liao et al.^61^ confirmed this finding by pointing out that a higher investment to GDP ratio is significantly related to the growth rate of energy consumption, with the average size effect being 0.08. To reverse this trend of declining GDP and escalating greenhouse gas levels, the region must focus on diversifying its energy mix and transitioning away from coal^75^. Structural change becomes imperative for the successful decarbonization of the regional economy^75^.

**Figure 3:**
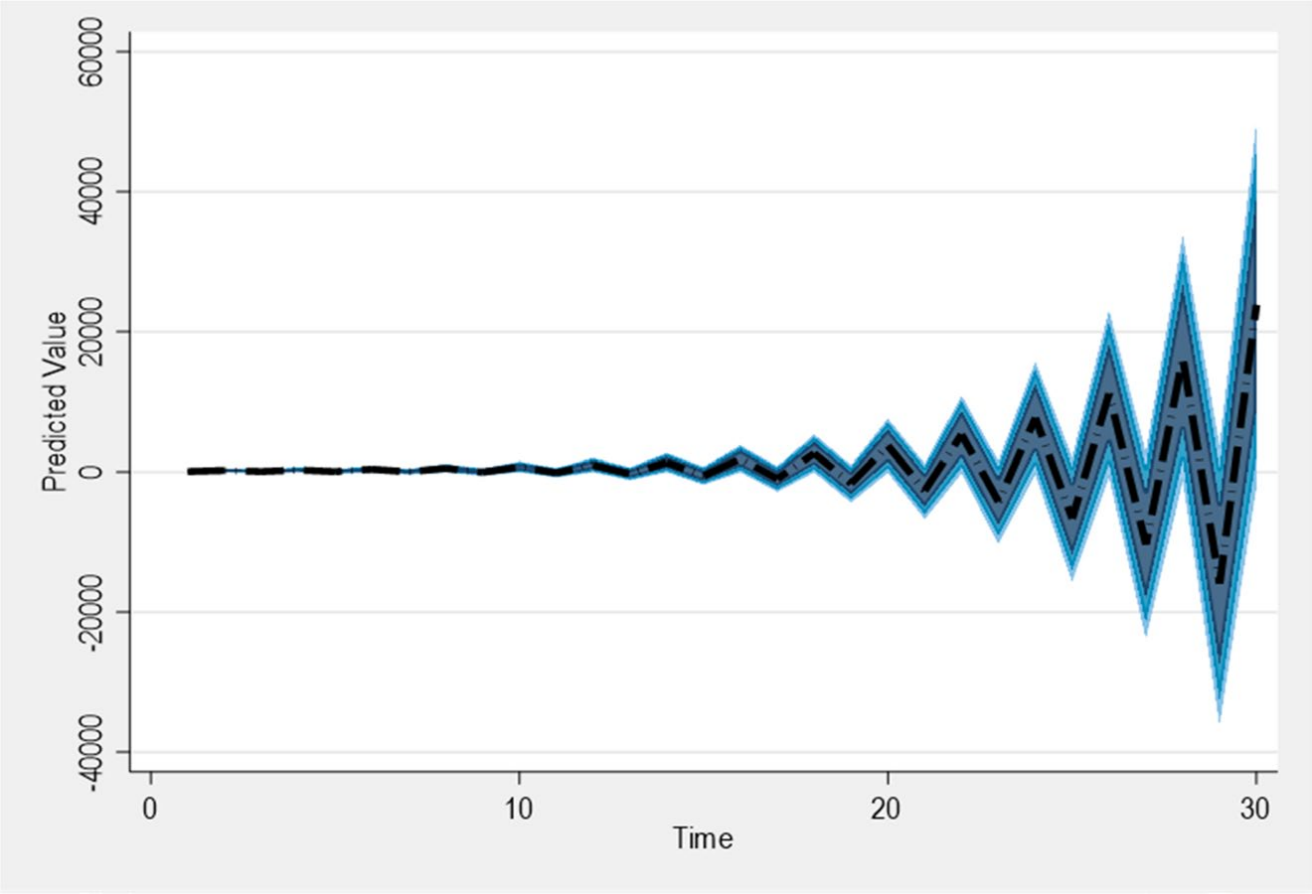
Plot generated using Dynardl and rarea option.

From the model of Table 6, the explained parameter did not appear as a lag form $t - 1$ but as a lagged first difference. The PPPIE no longer appears concurrently nonetheless as a first difference, whereas the rest of the variables appear as lagged and first difference forms. We estimate the lags $\left( {1,1,} \right)$, plus the laggdiffs $\left( {1,1,} \right)$. And then grow the number of simulations to five thousand. The output is shown in Fig. 3. Following the adverse economic shock at Time 10, there was a short-term decline in PPPIE, although not significantly different from the average forecast value. However, in response to negative shocks, PPPIE demonstrates growth, leading to a new forecast equilibrium beyond a time period of ten years. This anticipated growth indicates a positive trajectory for both investment and economic progress.

The dynardl approach applicable in conjunction with pssbound when fitting an error correction model. We performed pssbound as a post-estimation command for the cointegration test and represents in Table 1. The process automatically generated the F-statistics and t-statistics, the number of observations, and the number of variables^20^. From the analysis, we conclude that for the F statistic, there is no evidence of cointegration at the 5% level since the F statistic is 1.844 and above the critical value of I (1) 4.608. According to the F statistic, we did not detect any evidence of cointegration.

**Figure 4:**
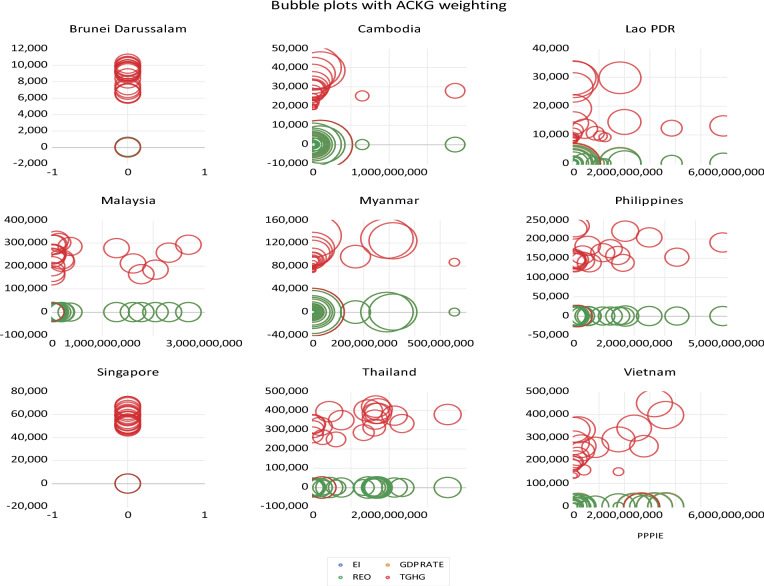
Bubble plots of variables.

A bubble plot (Fig. 4) is created to determine how well an estimation model fits the data and to identify the influencing relationship between variables. It shows the relationship between the variable effect and the moderator. The size of the bubble or mark reflects the accuracy of the study. The more precise the variable, the larger the bubble.

The bubble chart shows that Brunei Darussalam emits more amount of GHGs as it is the only more intense bubble there. In addition, Cambodia has many concentrated bubbles for REO in addition to GDPRATE and is one of the fastest-growing economies in Asia. In addition, People’s Democratic Republic of Laos also performs well on renewable electricity and emits more GHGs. The countries with significant economic growth, as well as REO are Malaysia, the Philippines, and Thailand. In contrast, Vietnam is an outlier in terms of economic growth rate (i.e., GDP), as well as REO and PPPIE. Vietnam’s economy grew at 8% in 2022, considered one of the fastest growing economies, and invested so much in offshore wind energy through auctions and tenders in PPPIE. Vietnam installed 779 MW of nearshore wind energy projects and was the third-largest offshore market in 2021^76^. Singapore is the only developed economy not producing significant renewable resources, and GDP growth rate is moderate. Its GHGs are growing significantly. Myanmar is also doing well in renewable power generation as this bubble becomes more concentrated^77^.

### Discussion

The analysis within the models reveals a noteworthy significance solely in GHGs concerning PPPIE. This finding signifies discernible contribution of private–public partnerships to heightened levels of GHGs in the ASEAN region. This observation resonates with broader apprehensions surrounding the Asia–Pacific region’s substantial impact on global energy consumption and greenhouse gas emissions^78,79^. The ensuing discussion underscores the region’s heavy dependence on fossil fuels, particularly coal, accentuating the imperative for an equitable transition towards renewable energy sources as a crucial step toward ensuring environmental sustainability^80^. Furthermore, the results also indicate a significant correlation between both GHGs and GDP growth rate, with a negative association identified for the latter. The prevalent reliance on fossil fuels in the ASEAN region manifests as a contributing factor to diminished economic development, reflected explicitly in the negative GDP growth rate. Additionally, the conspicuous surge in GHG emissions accentuates the environmental challenges intertwined with prevailing energy consumption patterns. The ensuing discourse underscores the urgency for diversifying the energy mix and steering away from coal to facilitate a successful and industrially viable decarbonization process. Collectively, these findings underscore the intricate interplay between private–public partnerships, economic progress, and the imperative for environmental sustainability within the ASEAN region. The discussion highlights the urgency of transitioning to cleaner energy sources, particularly given the susceptibility to external shocks like the COVID-19 pandemic. Recommendations stemming from these findings advocate for a strategic focus on diversification, increased investments in renewable energy, and fostering international collaborations to pave the way for a sustainable and resilient energy future in the region. Effectively addressing the challenges outlined in the models necessitates the formulation and implementation of a comprehensive strategy that seamlessly integrates economic development objectives with environmental considerations.

## Conclusion, policy recommendation, and limitation

### Conclusion

In conclusion, this paper delved into the impact of Public–private Partnership Investment in Energy (PPPIE) on the progress of ASEAN countries towards achieving SDG7 from 1999 to 2021. The research employed dynamic ARDL and bounds cointegration analyses, ensuring the stationarity of variables through Fisher-type unit-root tests based on Phillips–Perron tests. Notably, the study uncovered that a negative shock at time t = 10 had an immediate, albeit statistically insignificant, effect on reducing both GDP growth rate (GDPRATE) and PPPIE. However, over the same period, this shock led to a sustained decline in both variables, ultimately stabilizing at around 8 in the long term. The tangible example of the COVID-19 pandemic illustrated how such shocks could disrupt global economic growth. Moreover, the research identified a noteworthy negative relationship between GDP growth rate and PPPIE, indicating that PPPIE can decrease GDP growth rate by up to 8.3%. This underscores the detrimental impact of energy investments in the ASEAN region on the economy, posing potential risks to sustainability and hindering economic growth. The study highlights the prevalent trend in many developing nations, where energy infrastructure investments are primarily government-led, with limited involvement from the private sector, possibly explaining the observed negative correlation with PPPIE. Additionally, the energy investments in the ASEAN region exhibit predisposition towards fossil fuels, contributing significantly to a notable increase in greenhouse gas (GHG) emissions. Given that around 80% of energy consumption in the region is derived from fossil fuels, this heightened emissions level stands at 52.6%. The implication is that the current PPPIE does not support sustainable clean energy ambitions of the region. To sum up, the study highlights the complex interplay between public–private energy investments, GDP growth rate, and environmental sustainability in the ASEAN region. The findings underscore the need for a more balanced and sustainable approach to energy investments to mitigate adverse economic consequences and reduce GHG emissions. Additionally, exploring alternative and more sustainable energy investment models could be crucial for fostering economic growth without compromising environmental and economic stability.

### Policy recommendation

Consequently, the study recommends for the vital involvement of multilateral development banks, particularly highlighting the Asian Development Bank, in leading initiatives to stimulate significant investments in the Southeast Asian region. It’s noteworthy that the pursuit of SDG7 holds global significance beyond Southeast Asia. The expected increase in renewable energy adoption in the region, along with reservations about coal consumption, introduces significant considerations with far-reaching implications for national power grids and the environment. Specifically, policymakers should prioritize diversifying energy sources to enhance resilience to external shocks and promote sustainable development. In light of insightful policy propositions, the ASEAN region should implement the following action points: firstly, increase electricity supply to underserved communities by investing in on-grid and off-grid technologies that utilize renewable energy, extendable to remote areas. Secondly, implement policies and strategies supporting the use of clean cooking technologies, including electric stoves, liquefied petroleum gas, and sustainable bioenergy, reducing dependence on conventional biomass. Thirdly, promote the development of domestic renewable energy markets by providing policy and regulatory support and enhancing financing opportunities, particularly in the heating and transport sectors. Fourthly, strengthen regional and national grid infrastructure to enhance the reliability of modern services and accommodate intermittent renewable energy. Lastly, strengthen regional cooperation and knowledge sharing to improve sustainable energy security and increase the utilization of renewable energy through PPPIE.

### Limitation of the study

Finally, caution is advised for researchers and policymakers when interpreting these findings, given the inherent data limitations and contextual variations among individual countries in this study. Notably, certain countries, like Singapore, lack specific PPPIE data for certain time periods; however, these data gaps did not significantly influence the study’s outcomes. Future research should explore diverse regions in assessing progress toward SDG7. It’s crucial to recognize that the study’s conclusions are drawn from developing countries, limiting their direct applicability to developed nations, and the applied models have inherent limitations, particularly the assumption of a linear relationship in the ARDL model and potential impact from the choice of lag length.

## Data Availability

The data that support the findings of this study are openly available on request and was downloaded from https://databank.worldbank.org/source/world-development-indicators

## Abbreviations

ACKG: Access to clean cooking solutions
ARDL: Auto regression distributive lag
ASEAN: Association of Southeast Asian nations
COVID: Coronavirus disease
Dynardl: Dynamic auto regression distributive lag
D-h: Demetricus and Hurlin
EI: Energy intensity
ESG: Environmental, social, and governance
FGLS: Feasible generalized least squares
GDP: Gross domestic product
GECM: Generalized error correction model
GHG: Greenhouse gases
GCF: Global climate fund
ACGF: ASEAN catalytic green finance facility
GW: Gigawatts
KT: Kiloton
MJ: Metric joule
MMQR: Moment method of quantile regression
OECD: Organization for economic cooperation and development
Pssbound: Pesaran, Shin and Smith cointegration
PJ: Per joule
PPP: Purchasing power parity
PPPIE: Public private investment in energy
RE: Renewable energy
REO: Renewable electricity output
SDG: Sustainable development goal
SDS: Sustainable development scenario
SIBC: Schwarz’s Bayesian information criterion
STIRPAT: Stochastic impacts by regression on population, affluence, and technology
WHO: World Health Organization

## References

[1] IEA, IRENA, U. N. S. Division, W. Bank, & W. H. Organization. *Tracking SDG7: The energy progress report 2023*. (2023).

[2] Ning, L., Abbasi, K. R., Hussain, K., Alvarado, R. & Ramzan, M. Analyzing the role of green innovation and public–private partnerships in achieving sustainable development goals: A novel policy framework. *Environ. Sci. Pollut. Res.* (2023). 10.1007/s11356-023-26414-6 36964469

[3] IRENA & CPI*. Global landscape of renewable energy finance 2023*, International Renewable Energy Agency, Abu Dhabi. https://www.irena.org/-/media/Files/IRENA/Agency/Publication/2023/Feb/IRENA_CPI_Global_RE_finance_2023.pdf?rev=8668440314f34e588647d3994d94a785 (2023).

[4] Ahmed N, Areche FO, Sheikh AA, Lahiani A (2022). Green finance and green energy nexus in ASEAN countries: A bootstrap panel causality test. Energies.

[5] Caglar AE, Zafar MW, Bekun FV, Mert M (2022). Determinants of CO_2_ emissions in the BRICS economies: The role of partnerships investment in energy and economic complexity. Sustain. Energy Technol. Assess..

[6] Muganyi T, Yan L, Sun HP (2021). Green finance, fintech and environmental protection: Evidence from China. Environ. Sci. Ecotechnol..

[7] McCauley D, Pettigrew K (2023). Building a just transition in Asia-Pacific: Four strategies for reducing fossil fuel dependence and investing in clean energy. Energy Policy.

[8] Lup ANK, Soni V, Keenan B, Son J, Taghartapeh MR, Morato MM (2023). Sustainable energy technologies for the global south: Challenges and solutions toward achieving SDG 7. Environ. Sci. Adv..

[9] IRENA. Renewable energy outlook for ASEAN: Towards a regional energy transition. 2nd Edn, International Renewable Energy Agency, Abu Dhabi 978-92-9260-467-7 (2022).

[10] Banga J (2019). The green bond market: A potential source of climate finance for developing countries. J. Sustain. Finan. Invest..

[11] Baiashvili, T. & Gattini, L. Impact of FDI on economic growth: The role of country income levels and institutional strength. https://www.exploring-economics.org/en/discover/impact-of-fdi-on-economic-growth-the-role-of-co/ (2020).

[12] Hoa PX, Xuan VN, Thu NTP (2024). Determinants of renewable energy consumption in the fifth technology revolutions: Evidence from ASEAN countries. J. Open Innov. Technol. Market Complex..

[13] Vakulchuk R, Overland I, Suryadi B (2023). ASEAN’s energy transition: How to attract more investment in renewable energy. Energy Ecol. Environ..

[14] Kim, T. Y. & Karpinski, M. *Clean energy progress after the Covid-19 crisis will need reliable supplies of critical minerals.* International Energy Agency. https://www.iea.org/articles/clean-energy-progress-after-the-covid-19-crisis-will-need-reliable-supplies-of-critical-minerals (2020).

[15] Sakti AD, Rohayani P, Izzah NA, Toya NA, Hadi PO, Octavianti T (2023). Spatial integration framework of solar, wind, and hydropower energy potential in Southeast Asia. Sci. Rep..

[16] Maino AG (2022). Financing the Energy Transition : The Role, Opportunities and Challenges of Green Bonds.

[17] RISE. Energy Sector Management Assistance Program, 2020. Regulatory Indicators for Sustainable Energy 2020: Sustaining the Momentum, Washington (2020).

[18] Sumarno T, Fitriyanti V, Khusna V, Yusgiantoro I (2023). The importance of women participation in ensuring justice in energy transition in ASEAN and G7. Int. Conf. Gender Res..

[19] BANKTRACK. Banking on climate change-fossil fuel finance report card 2019. (2019).

[20] Jordan S, Philips AQ (2018). Cointegration testing and dynamic simulations of autoregressive distributed lag models. Stata J..

[21] da Silva PP, Cerqueira PA, Ogbe W (2018). Determinants of renewable energy growth in Sub-Saharan Africa: Evidence from panel ARDL. Energy.

[22] Wang J, Shahbaz M, Dong K, Dong X (2023). Renewable energy transition in global carbon mitigation: Does the use of metallic minerals matter?. Renew. Sustain. Energy Rev..

[23] PWC. Energy transition readiness in Southeast Asia. (2021).

[24] Shi J, Liu Y, Sadowski BM, Alemzero D, Dou S, Sun H (2023). The role of economic growth and governance on mineral rents in main critical minerals countries. Resour. Policy.

[25] Dou S, Xu D, Zhu Y, Keenan R (2023). Critical mineral sustainable supply: Challenges and governance. Futures.

[26] Sarpong KA, Xu W, Gyamfi BA, Ofor EK (2023). A step towards carbon neutrality in E7: The role of environmental taxes, structural change, and green energy. J. Environ. Manag..

[27] Durani F, Bhowmik R, Sharif A, Anwar A, Syed QR (2023). Role of economic uncertainty, financial development, natural resources, technology, and renewable energy in the environmental Phillips curve framework. J. Clean. Prod..

[28] Jahanger A, Awan A, Anwar A, Adebayo TS (2023). Greening the Brazil, Russia, India, China and South Africa (BRICS) economies: Assessing the impact of electricity consumption, natural resources, and renewable energy on environmental footprint. Nat. Resour. Forum.

[29] Liu B, Guan Y, Shan Y, Cui C, Hubacek K (2023). Emission growth and drivers in Mainland Southeast Asian countries. J. Environ. Manag..

[30] Yin S (2023). Decadal changes in premature mortality associated with exposure to outdoor PM2.5 in mainland Southeast Asia and the impacts of biomass burning and anthropogenic emissions. Sci. Total Environ..

[31] Ozili, P. K. & Iorember, P. T. Financial stability and sustainable development. *Int. J. Financ. Econ.* (2023).

[32] Emmerling, J., Aleluia Reis, L., Drouet, L., Raitzer, D. & Pradhananga, M. Assessing the implications of a global net-zero transition for developing Asia: Insights from Integrated assessment modeling. *Asian Development Bank Economics Working Paper Series,* (2023).

[33] Pereira L, Asrar GR, Bhargava R, Fisher LH, Hsu A, Jabbour J (2021). Grounding global environmental assessments through bottom-up futures based on local practices and perspectives. Sustain. Sci..

[34] UNESCAP, IEA, & IRENA. Advancing SDG7 in Asia and the Pacific. (2023).

[35] Li D, Bae JH, Rishi M (2022). Sustainable development and SDG-7 in Sub-Saharan Africa: Balancing energy access, economic growth, and carbon emissions. Eur. J. Dev. Res..

[36] Priyan S, Matahen R, Priyanshu D, Mouqdadi M (2024). Environmental strategies for a healthcare system with green technology investment and pandemic effects. Innov. Green Dev..

[37] Formisano V, Iannucci E, Fedele M, Bonab AB (2022). City in the loop: Assessing the relationship between circular economy and smart sustainable cities. Sinerg. Italian J. Manag..

[38] Bashir MF, Pan Y, Shahbaz M, Ghosh S (2023). How energy transition and environmental innovation ensure environmental sustainability? Contextual evidence from Top-10 manufacturing countries. Renew. Energy.

[39] Voumik LC, Ridwan M, Rahman MH, Raihan A (2023). An investigation into the primary causes of carbon dioxide releases in Kenya: Does renewable energy matter to reduce carbon emission?. Renew. Energy Focus.

[40] Danish, Ulucak R (2020). How do environmental technologies affect green growth? Evidence from BRICS economies. Sci. Total Environ..

[41] Lai F, Zhou J, Lu L, Hasanuzzaman M, Yuan Y (2023). Green building technologies in Southeast Asia: A review. Sustain. Energy Technol. Assess..

[42] Jiang Y, Sharif A, Anwar A, The Cong P, Lelchumanan B, Thi Yen V (2023). Does green growth in E-7 countries depend on economic policy uncertainty, institutional quality, and renewable energy? Evidence from quantile-based regression. Geosci. Front..

[43] Siddik AB, Khan S, Khan U, Yong L, Murshed M (2023). The role of renewable energy finance in achieving low-carbon growth: Contextual evidence from leading renewable energy-investing countries. Energy.

[44] Pan Y, Dong F (2023). Factor substitution and development path of the new energy market in the BRICS countries under carbon neutrality: Inspirations from developed European countries. Appl. Energy.

[45] Diesendorf M, Taylor R, Diesendorf M, Taylor R (2023). Transitioning the Energy System. The Path to a Sustainable Civilisation: Technological, Socioeconomic and Political Change.

[46] White, K., Loughead, R., Linstaedt, J., Rooze, J. & Young, W. Financing the transition: Energy supply investment and bank financing activity comparing low-carbon and fossil fuel activity: Summary report 1. (2023).

[47] Amundi and IFC. Emerging Market Green Bonds Report 2020: On the road to green recovery. Washington (2021).

[48] IEA. Southeast Asia Energy Outlook 2022. (2022).

[49] Yu H, Wang J, Hou J, Yu B, Pan Y (2023). The effect of economic growth pressure on green technology innovation: Do environmental regulation, government support, and financial development matter?. J. Environ. Manag..

[50] Samour A, Adebayo TS, Agyekum EB, Khan B, Kamel S (2023). Insights from BRICS-T economies on the impact of human capital and renewable electricity consumption on environmental quality. Sci. Reports.

[51] Devine, H. *et al.**Private finance for development wishful thinking or thinking out of the box?* International Monetary Fund. https://www.imf.org/en/Publications/Departmental-Papers-Policy-Papers/Issues/2021/05/14/Private-Finance-for-Development-50157 (2021).

[52] Glemarec Y (2023). Financing green and climate resilient infrastructure in ASEAN countries. Environ. Progress Sustain. Energy.

[53] Philips AQ (2018). Have your cake and eat it too? Cointegration and dynamic inference from autoregressive distributed lag models. Am. J. Politic. Sci..

[54] Williams LK, Whitten GD (2011). Dynamic simulations of autoregressive relationships. Stata J..

[55] Anteneh GD, Andries FH, Paul LL, Detlef PVV (2019). Scenario analysis for promoting clean cooking in Sub-Saharan Africa: Costs and benefits. Energy.

[56] Rosenthal J, Quinn A, Grieshop AP, Pillarisetti A, Glass RI (2018). Clean cooking and the SDGs: Integrated analytical approaches to guide energy interventions for health and environment goals. Energy Sustain. Dev..

[57] Sumarno, T., Fitriyanti, V., Khusna, V. & Yusgiantoro, I. The importance of women participation in ensuring justice in energy transition in ASEAN and G7. Proceedings of the 6th International Conference on Gender Research **6**, (2023).

[58] Du G (2023). Nexus between green finance, renewable energy, and carbon intensity in selected Asian countries. J. Clean. Prod..

[59] Ilyas M, Mu Z, Akhtar S, Hassan H, Shahzad K, Aslam B (2024). Renewable energy, economic development, energy consumption and its impact on environmental quality: New evidence from South East Asian countries. Renew. Energy.

[60] Hussain ZH, Mehmood B, Khan MK, Tsimisarak RSM (2022). Green growth, green technology, and environmental health: Evidence from High-GDP countries. Front. Public Health.

[61] Liao H, Peng Y, Wang F, Zhang T (2022). Understanding energy use growth: The role of investment-GDP ratio. Struct. Change Econ. Dyn..

[62] Sikder M, Wang C, Yao X, Huai X, Wu L, KwameYeboah F (2022). The integrated impact of GDP growth, industrialization, energy use, and urbanization on CO2 emissions in developing countries: Evidence from the panel ARDL approach. Sci. Total Environ..

[63] Agusdinata DB, Liu W (2023). Global sustainability of electric vehicles minerals: A critical review of news media. Extract. Ind. Soc..

[64] Williams B, Bishop D, Hooper G, Chase JG (2024). Driving change: Electric vehicle charging behavior and peak loading. Renew. Sustain. Energy Rev..

[65] del Río JML, López ER, Fernández J (2024). Tribological behavior of electric vehicle transmission oils using Al2O3 nanoadditives. J. Mol. Liq..

[66] Narayan PK (2005). The saving and investment nexus for China: Evidence from cointegration tests. Appl. Econ..

[67] Pesaran MH, Shin Y, Smith RJ (2001). Bounds testing approaches to the analysis of level relationships. J. Appl. Econ..

[68] Aboagye PD, Sharifi A (2024). Urban climate adaptation and mitigation action plans: A critical review. Renew. Sustain. Energy Rev..

[69] Qiu J, Seah S, Martinus M (2024). Examining climate ambition enhancement in ASEAN countries’ nationally determined contributions. Environ. Dev..

[70] Wu H, Feng Z, Sun T, Li R, Zhao H (2024). Efficiency, sustainability, and resilience a trifecta for a green economic recovery through natural resource markets. Resour. Policy.

[71] Raza SA, Siddiqui AW (2024). Exploring temporal demand patterns of refined petroleum products: Implications of the COVID-19 pandemic as a black swan event. Extract. Ind. Soc..

[72] Hu Y, Weng L (2024). Net-zero energy transition in ASEAN countries: The evolutionary model brings novel perspectives to the cooperative mechanism of climate governance. J. Environ. Manag..

[73] IEA. World Energy Investment. (2020).

[74] Naumann G, Schropp E, Steegmann N, Möller MC, Gaderer M (2024). Environmental performance of a hybrid solar-hydrogen energy system for buildings. Int. J. Hydrog. Energy.

[75] Wahab S, Imran M, Ahmed B, Rahim S, Hassan T (2024). Navigating environmental concerns: Unveiling the role of economic growth, trade, resources and institutional quality on greenhouse gas emissions in OECD countries. J. Clean. Prod..

[76] Elsner P (2019). Continental-scale assessment of the African offshore wind energy potential: Spatial analysis of an under-appreciated renewable energy resource. Renew. Sustain. Energy Rev..

[77] Shi J, Hu X, Dou S, Alemzero D, Alhassan EA (2023). Evaluating technological innovation impact. An empirical analysis of the offshore wind sector. Environ. Sci. Pollut. Res..

[78] Iyke BN (2024). Climate change, energy security risk, and clean energy investment. Energy Econ..

[79] Pi K, Khan S, Raza SA, Shahzadi I (2024). Sustainable energy efficiency, greener energy and energy-related emissions nexus: Sustainability-related implications for G7 economies. Geol. J..

[80] Caglar AE, Daştan M, Rej S (2024). A new look at China’s environmental quality: how does environmental sustainability respond to the asymmetrical behavior of the competitive industrial sector?. Int. J. Sustain. Dev. World Ecol..

